# Supplementary material for: Evolutionary Dynamics of Chloroplast Genome and Codon Usage in the Genus Diospyros (Ebenaceae)
Source: Biology (Basel). 2025 Nov 9;14(11):1568. doi: 10.3390/biology14111568 (PMC12650415; doi:10.3390/biology14111568)

Supplementary Information

Table S1. List of gene contents in the plastome of *Diospyros tsangii.*

Table S2. Types and numbers of SSRs within the chloroplast genomes of fifteen *Diospyros* species.

Table S3. Types and numbers of the repeat sequences in fifteen *Diospyros* species.

Table S4. The *dN*/*dS* values of CDS genes within fifteen species of the *Diospyros* genus.

Table S5. Genes used for codon usage bias analysis within the chloroplast genomes of fifteen *Diospyros* species.

Figure S1. Chloroplast genome map of *D. tsangii.*

Figure S2. Collinearity analysis of the chloroplast genomes of fifteen *Diospyros* species.

Figure S3. Evolutionary pressure assessment of plastid gene orthologs across 15 *Diospyros* species .

Figure S4. The RSCU of amino acids in 15 species chloroplast genomes within the genus *Diospyros*.

Figure S5. Neutral-plot analysis in 15 species chloroplast genomes within the genus *Diospyros.*

Figure S6. ENC-plot analysis in 15 species chloroplast genomes within the genus *Diospyros*.

Figure S7. Distribution of ENC ratio in 15 species chloroplast genomes within the genus *Diospyros*.

Figure S8. PR2-plot analysis in 15 species chloroplast genomes within the genus *Diospyros*.

Table S1. List of gene contents in the plastome of *Diospyros tsangii.*

Category	Groups of Genes	Names of Genes
Self-replication	Ribosomal RNA	*rrn4.5*[*], *rrn5*[*], *rrn16*[*], *rrn23*[*]
	Transfer RNA	*trnA-UGC*[*,a], *trnC-GCA*, *trnD-GUC*, *trnE-UUC*, *trnF-GAA*, *trnM-CAU*[*], *trnG-GCC*, *trnG-UCC*[a], *trnH-GUG*, *trnI-GAU*[*,a], *trnI-CAU*[*], *trnK-UUU*[a], *trnL-CAA*[*], *trnL-UAA*[a], *trnL-UAG, trnN-GUU*[*], *trnP-UGG*, *trnQ-UUG*, *trnR-ACG*[*], *trnR-UCU*, *trnS-GCU*, *trnS-GGA*, *trnS-UGA*, *trnT-GGU*, *trnT-UGU*, *trnV-GAC*[*], *trnV-UAC*[a], *trnW-CCA*, *trnY-GUA*
	Small subunit of ribosome	*rps2*, *rps3*, *rps4*, *rps7*[*], *rps8*, *rps11*, *rps12*[*,b], *rps14*, *rps15*, *rps16*, *rps18*, *rps19*
	Large subunit of ribosome	*rpl2*[*,a], *rpl14*, *rpl16*[a], *rpl20*, *rpl22*, *rpl23*[*], *rpl32*, *rpl33*, *rpl36*
	RNA polymerase subunit	*rpoA*, *rpoB*, *rpoC1*[a], *rpoC2*
Photosynthesis	ATP synthase	*atpA*, *atpB*, *atpE*, *atpF*[a], *atpH*, *atpI*
	NADH dehydrogenase	*ndhA*[a], *ndhB*[*,a], *ndhC*, *ndhD*, *ndhE*, *ndhF*, *ndhG*, *ndhH*, *ndhI*, *ndhJ*, *ndhK*
	Cytochrome b/f complex	*petA*, *petB*[a], *petD*[a], *petG*, *petL*, *petN*
	PhotosystemI	*psaA*, *psaB*, *psaC*, *psaI*, *psaJ*
	PhotosystemII	*psbA*, *psbB*, *psbC*, *psbD*, *psbE*, *psbF*, *psbH*, *psbI*, *psbJ*, *psbK*, *psbL*, *psbM*, *psbN*, *psbT*, *psbZ*
	Rubisco large subunit	*rbcL*
Others	Translation initiation	*infA*
	Proteolysis	*clpP*[b]
	Cytochrome *c* biogenesis protein	*ccsA*
	Acetyl-CoA carboxylase	*accD*
	Chloroplast envelope membrane protein	*cemA*
	maturase	*matK*
	Hypothetical reading frame	*ycf1*, *ycf2*[*], *ycf3*[b], *ycf4*, *ycf15*[*]

*, duplicated gene in IR; [a], gene including one intron; [b],gene including two introns.

Table S2. Types and numbers of SSRs within the chloroplast genomes of fifteen *Diospyros* species.

Species	SSR Type	Mono		Di	Tri		Tetra					Penta	
	No.	A / T	C / G	AT / AT	AAT / ATT	AGC / CTG	AAAG / CTTT	AAAT / ATTT	AATC / ATTG	AATT / AATT	AGAT / ATCT	AAACT / AGTTT	AAAAT / ATTTT
D. oleifera	71	59	0	3	1	1	1	5	1	0	0	0	0
D. tsangii	72	60	0	3	1	1	1	5	1	0	0	0	0
D. kaki	70	59	0	3	1	1	1	4	1	0	0	0	0
D. vaccinioides	72	60	0	3	1	1	1	5	1	0	0	0	0
D. glaucifolia	77	63	3	3	1	1	1	4	1	0	0	0	0
D. lotus	76	64	1	3	1	1	1	4	1	0	0	0	0
D. morrisiana	66	53	1	3	1	1	1	5	1	0	0	0	0
D. maclurei	57	42	0	3	3	1	1	3	2	0	1	1	0
D.hainanensis	53	40	1	3	0	1	1	5	1	0	0	1	0
D. strigosa	53	44	0	2	1	0	0	4	1	0	0	1	0
D. eriantha	53	44	0	2	1	0	0	4	1	0	0	1	0
D. dumetorum	71	55	0	5	1	0	0	5	1	2	0	0	2
D. rhombifolia	48	29	1	5	1	1	2	6	1	1	0	1	0
D. cathayensis	48	29	0	5	2	1	2	6	1	1	0	1	0
D. sutchuensis	51	34	1	4	2	1	1	6	1	0	0	1	0

Table S3. Types and numbers of the repeat sequences in fifteen *Diospyros* species.

Species	Forward repeat	Reverse repeat	Palindromic repeat	Complement repeat
D. oleifera	24	2	24	0
D. tsangii	21	2	27	0
D. kaki	15	1	34	0
D. vaccinioides	24	3	23	0
D. glaucifolia	24	1	25	0
D. lotus	23	1	26	0
D. morrisiana	23	4	23	0
D. maclurei	27	1	21	1
D. hainanensis	25	0	25	0
D. strigosa	23	2	25	0
D. eriantha	22	1	27	0
D. dumetorum	19	6	24	1
D. rhombifolia	21	1	28	0
D.cathayensis	22	2	26	0
D. sutchuensis	23	2	25	0

Table S4. The *dN*/*dS* values of CDS genes within fifteen species of the *Diospyros* genus.

Gene category	Genes	Whole
Self-replication	*rpl2*	0.7144287886086300
	rpl14	0.0795045578690700
	rpl16	0.2015311644192100
	rpl20	0.1482900597039300
	rpl22	0.2872420464329100
	rpl23	0.0000000000000000
	rpl32	0.1370461105709100
	rpl33	0.7375545167570600
	rpl36	0.0000000000000000
	rps2	0.1705749226960500
	rps3	0.1694722314481900
	rps4	0.7654896594293200
	rps7	0.0000000000000000
	rps8	0.3347186410586700
	rps11	0.0853797546309500
	rps12	0.0000000000000000
	rps14	0.4958820364196300
	rps15	0.6359212897408100
	rps16	0.4605589964973300
	rps18	0.4329553282663800
	rps19	0.2356970084240900
	rpoA	0.3317774625053100
	rpoB	0.1921345253042800
	rpoC1	0.1535059704885400
	rpoC2	0.2416201862906600
Photosynthesis	*atpA*	0.0909820955170500
	atpB	0.2457921563219600
	atpE	0.3431704890656500
	atpF	0.2522763879233900
	atpH	0.0000000000000000
	atpI	0.1367598091854300
	ndhA	0.1116694189145700
	ndhB	0.2633193189914500
	ndhC	0.1823612725958800
	ndhD	0.2560292058827000
	ndhE	0.1178192343867800
	ndhF	0.1441527349603500
	ndhG	0.1714625659039700
	ndhH	0.0958362338320000
	ndhI	0.0000000000000000

	ndhJ	0.1560510265765100
	ndhK	0.1441292921003900
	petA	0.3408266370643400
	petB	0.3499497902960400
	petD	0.4055225084437000
	petG	0.5035296028428600
	petL	0.0000000000000000
	petN	0.0000000000000000
	psaA	0.0917501582150100
	psaB	0.0790747019525800
	psaC	0.0000000000000000
	psaI	0.0000000000000000
	psaJ	0.4893976994536600
	psbA	0.1297853825445400
	psbB	0.0525307687376100
	psbC	0.1160686090950400
	psbD	0.1315212161389200
	psbE	0.3820681797584100
	psbF	0.0000000000000000
	psbH	0.0000000000000000
	psbI	1.0010282288124700
	psbJ	0.2673991410623800
	psbK	0.3278490692531800
	psbL	0.0000000000000000
	psbM	0.0000000000000000
	psbN	0.0000000000000000
	psbT	0.4921631138908200
	psbZ	0.2482981944812500
	rbcL	0.5486293224379700
**Other**	*accD*	0.1859545238541900
	ccsA	0.3005590522721000
	cemA	0.3139569561778700
	clpP	0.4064056726721100
	infA	0.3791759089860100
	matK	0.3684673765939200
	ycf1	0.3446208175347500
	ycf2	0.3425119281058600
	ycf3	0.3089908916963200
	ycf4	0.2731791639256700
	ycf15	0.3964841929304200

Table S5. Genes used for codon usage bias analysis within the chloroplast genomes of fifteen *Diospyros* species.

**Gene list**
accD, *atpA*, *atpB*, *atpE*, *atpF*, *atpI*, *ccsA*, *cemA*, *clpP*, *matK*, *ndhA*, *ndhB*, *ndhC*, *ndhE*, *ndhF*, *ndhG*, *ndhH*, *ndhI*, *ndhJ*, *ndhK*, *petA*, *petB*, *petD*, *psaA*, *psaB*, *psbA*, *psbB*, *psbC*, *psbD*, *rbcL*, *rpl2*, *rpl14*, *rpl16*, *rpl20*, *rpl22*, *rpoA*, *rpoB*, *rpoC1*, *rpoC2*, *rps11*, *rps12*, *rps14*, *rps18*, *rps2*, *rps3*, *rps4*, *rps7*, *rps8*, *ycf1*, *ycf2*, *ycf3*, *ycf4*

Figure S1. Chloroplast genome map of *D. tsangii*. Genes positioned on the outer circle are transcribed in a counterclockwise direction, whereas those on the inner circle are transcribed clockwise. Within the inner circle, dark gray regions indicate segments with elevated GC content, while lighter gray areas correspond to regions with higher AT content.

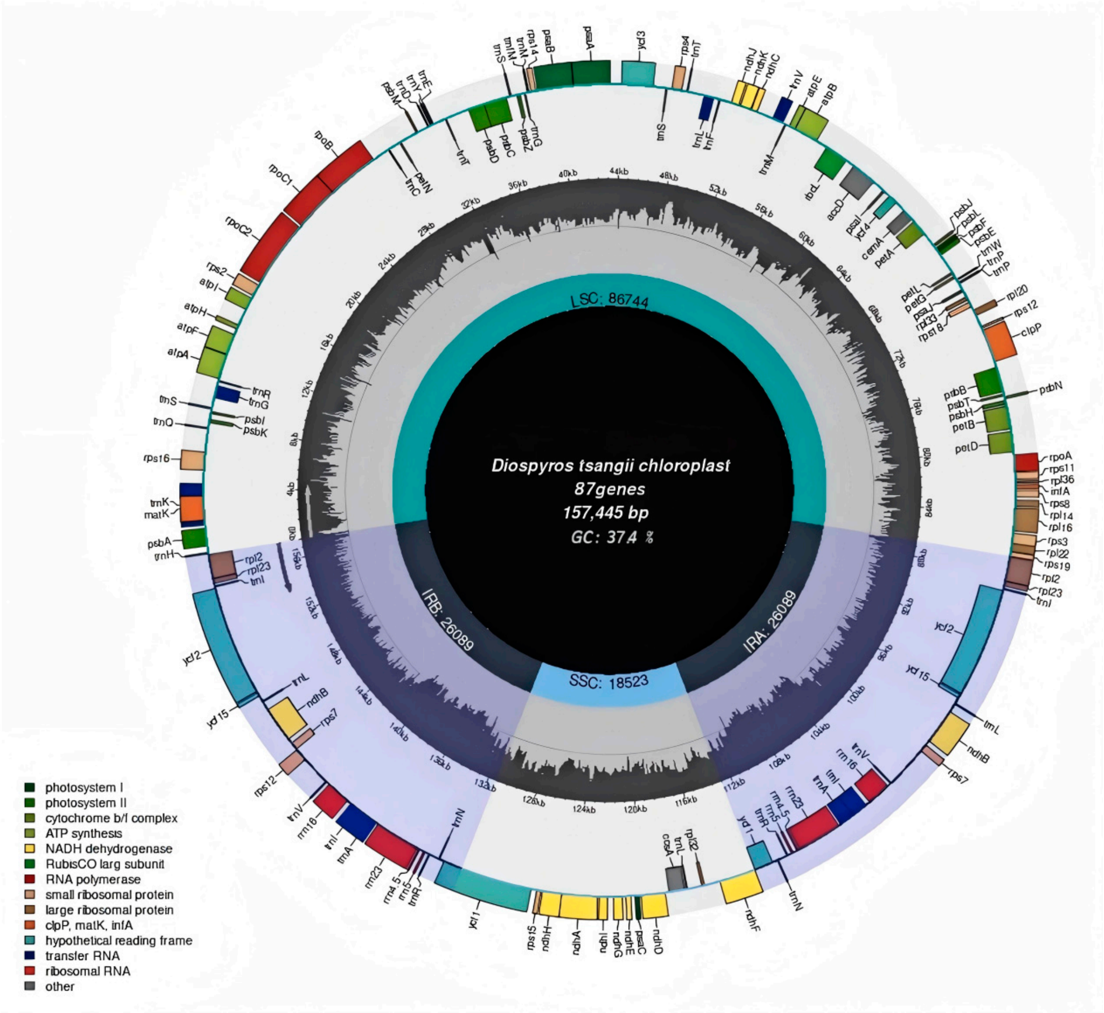

Figure S2. Colinearity analysis of the chloroplast genomes of fifteen *Diospyros* species.

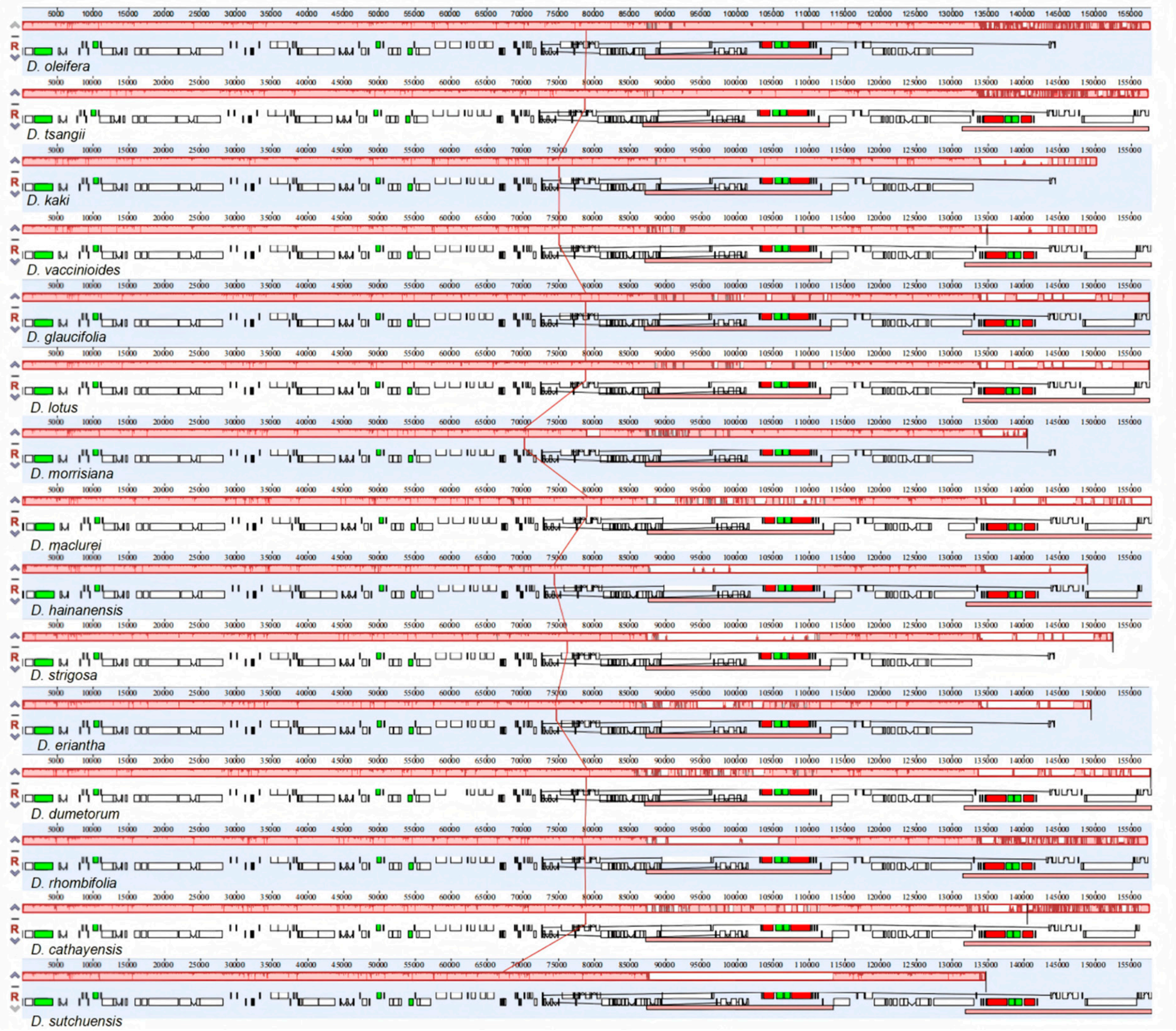

Figure S3. Evolutionary pressure assessment of plastid gene orthologs across 15 *Diospyros* species.

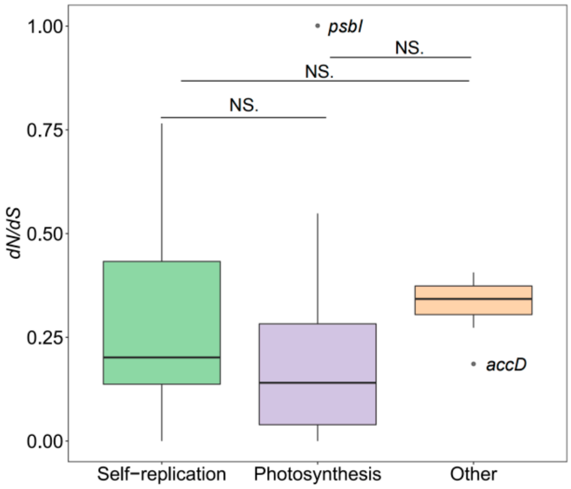

Figure S4. The RSCU of amino acids in 15 species chloroplast genomes within the genus *Diospyros*.

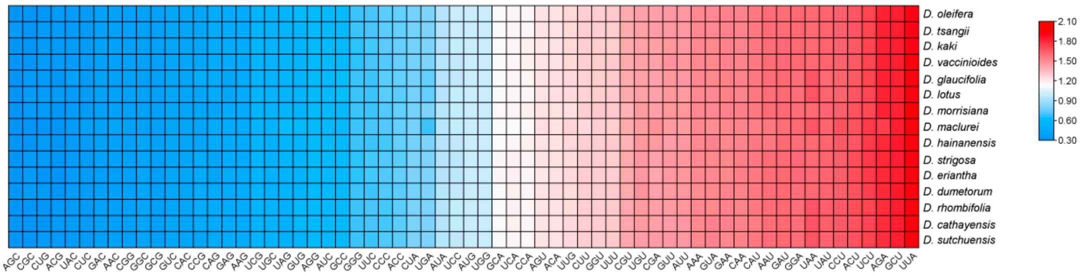

Figure S5. Neutral-plot analysis in 15 species chloroplast genomes within the genus *Diospyros.* The black line in the plot illustrates the correlation trend, with the corresponding equation displayed at the bottom of the graph.

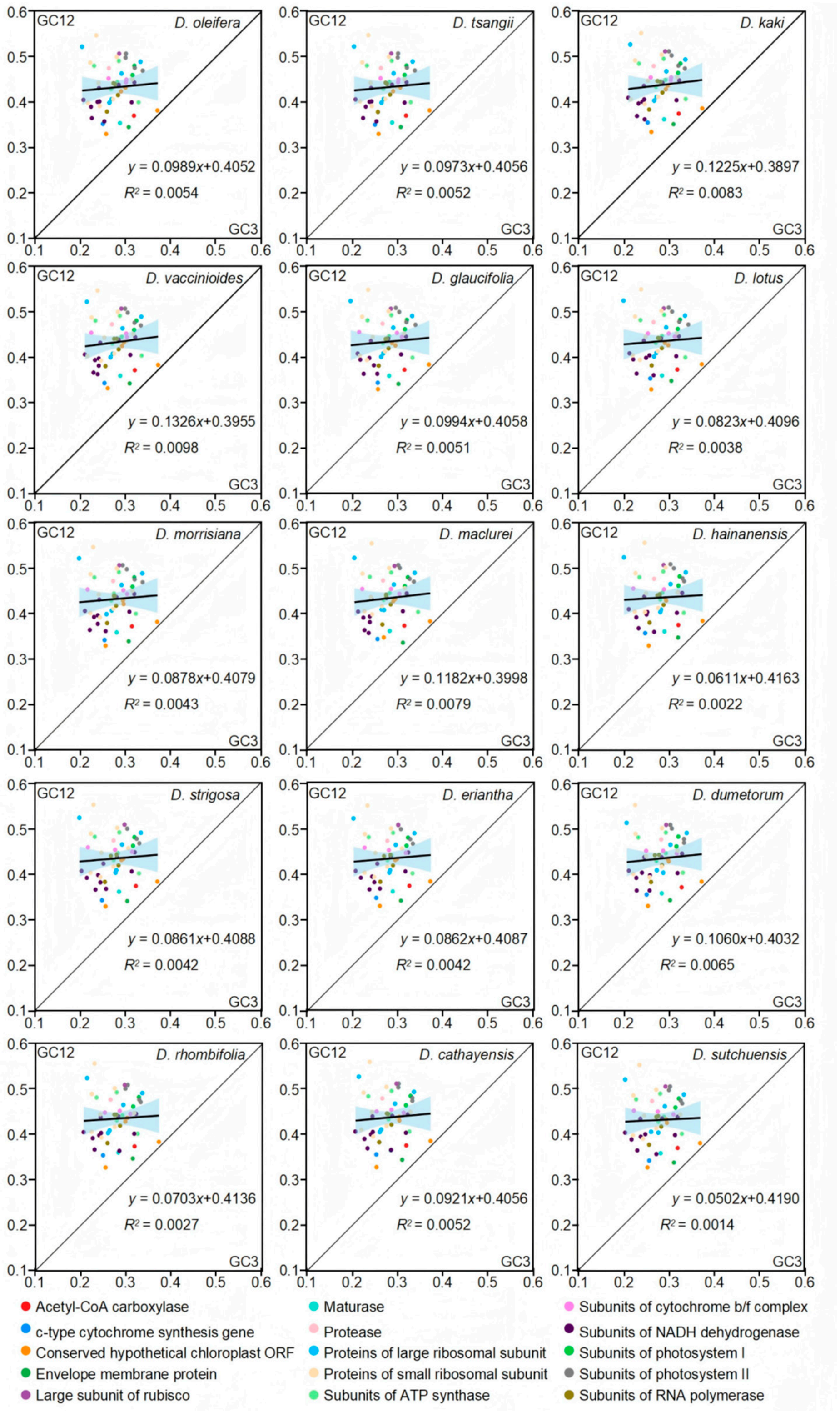

Figure S6. ENC-plot analysisin 15 species chloroplast genomes within the genus *Diospyros*. If a data point is significantly distant from the standard curve, this indicates that the codon usage bias of chloroplast coding sequences is predominantly influenced by natural selection.

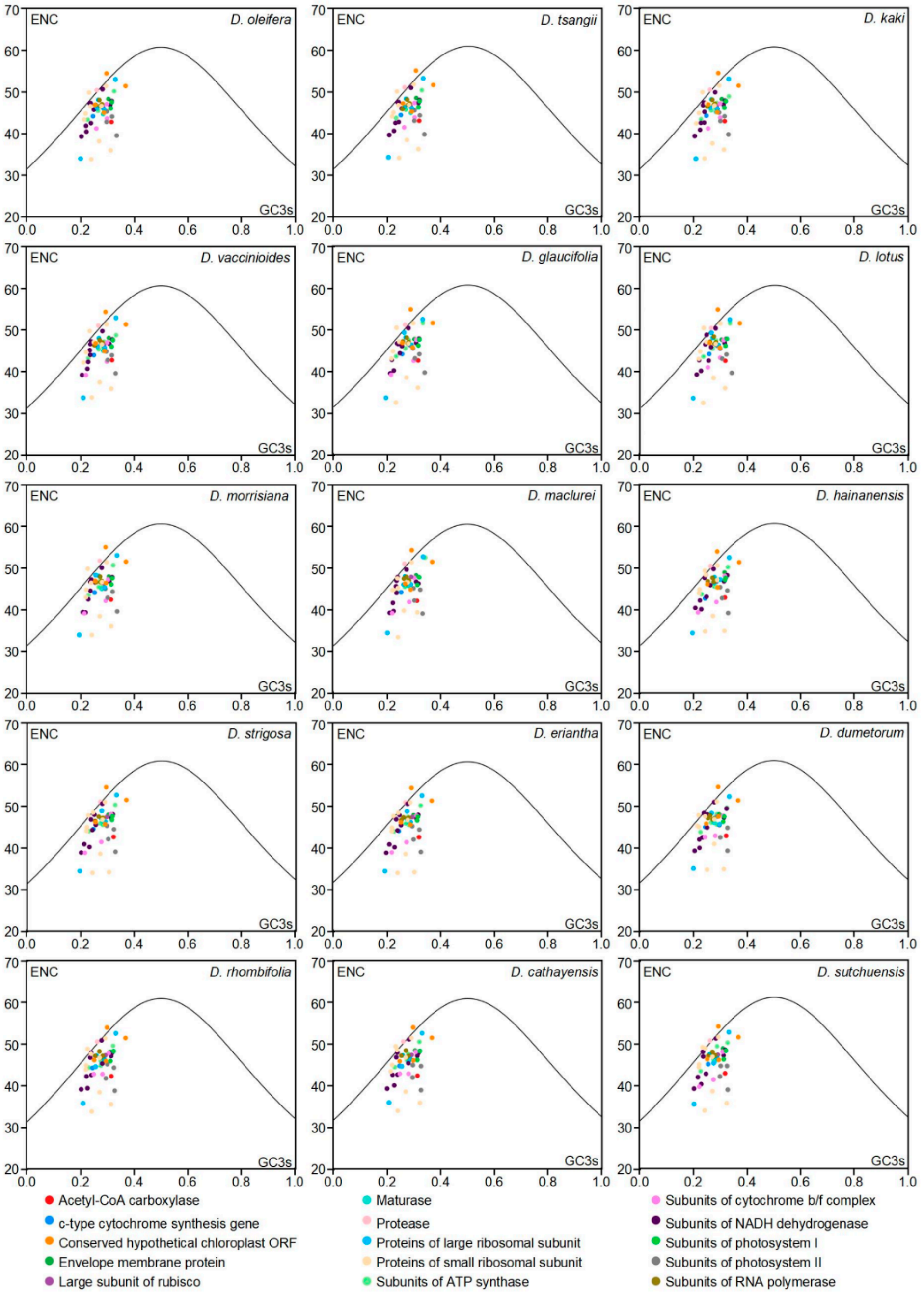

Figure S7. Distribution of ENC ratio in 15 species chloroplast genomes within the genus *Diospyros*.

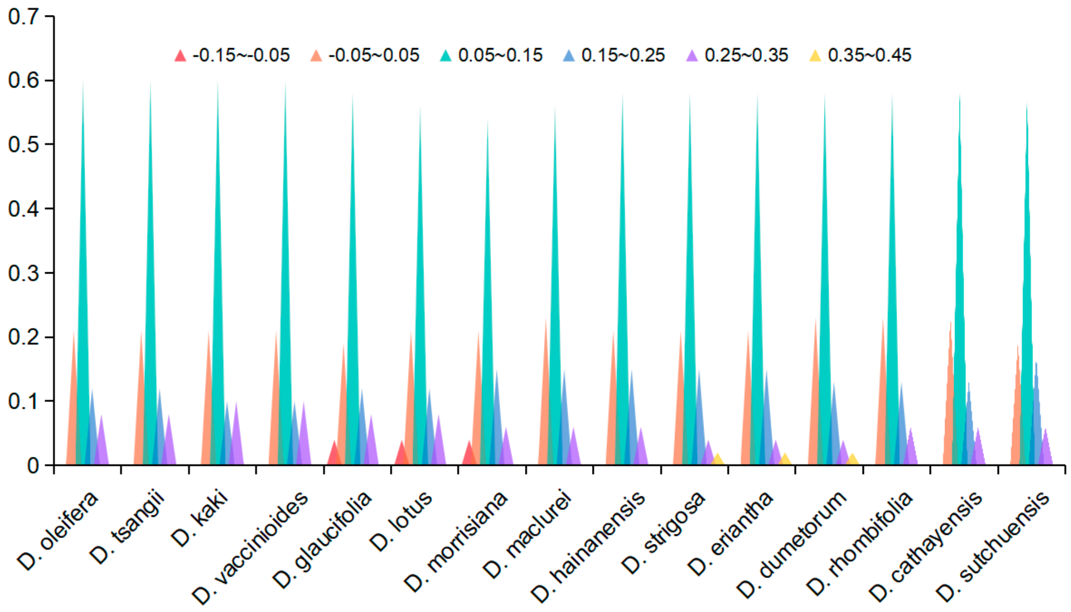

Figure S8. PR2-plot analysis in 15 species chloroplast genomes within the genus *Diospyros*.

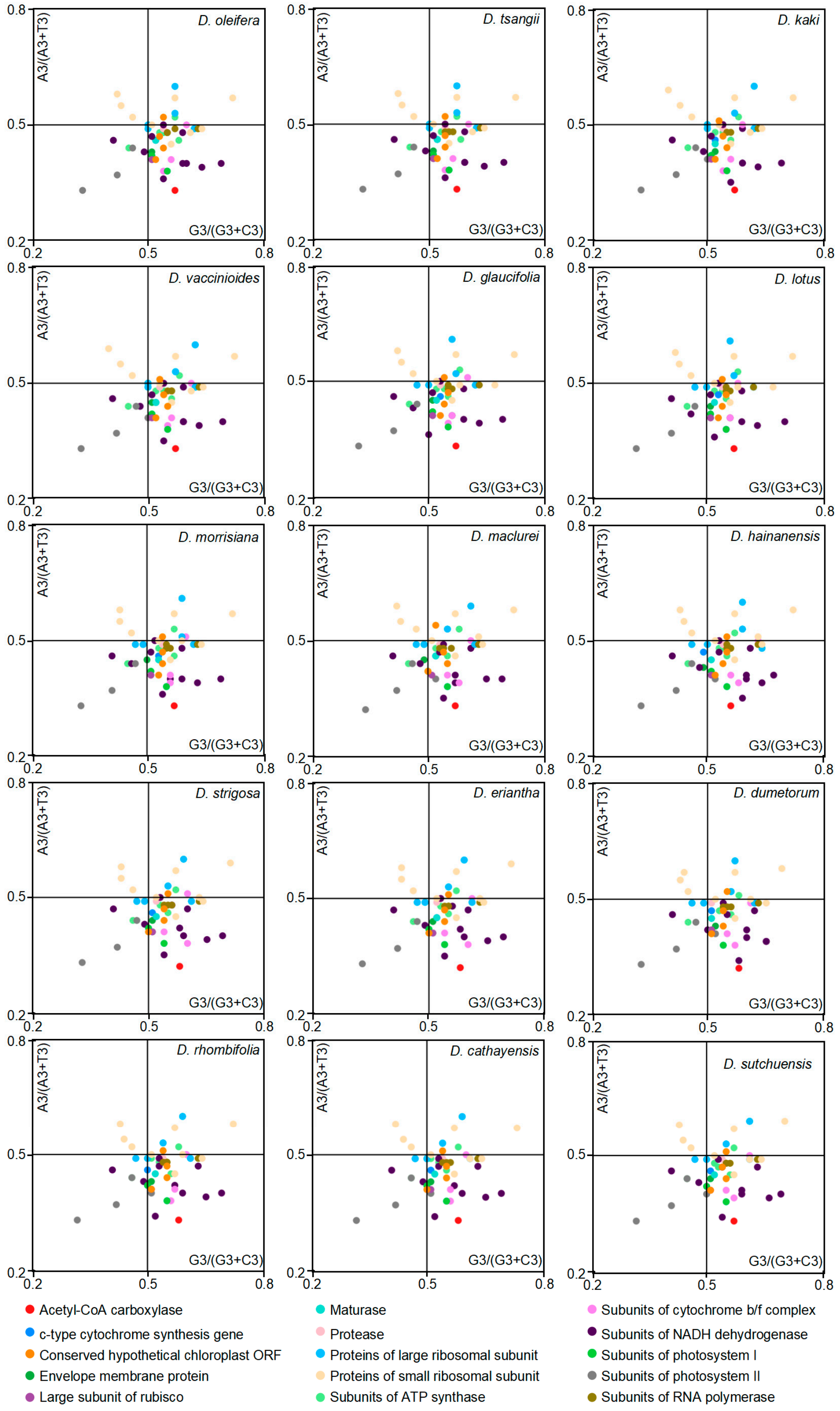

Supplement: Supplementary file 1 [file biology-14-01568-s001.zip › biology-3960748-supplementary.pdf]
